# Supplementary material for: Jagged1-Notch1-deployed tumor perivascular niche promotes breast cancer stem cell phenotype through Zeb1
Source: Nat Commun. 2020 Oct 12;11:5129. doi: 10.1038/s41467-020-18860-4 (PMC7552407; doi:10.1038/s41467-020-18860-4)
Supplement: Supplementary file 1 — Supplementary Information [file 41467_2020_18860_MOESM1_ESM.pdf]

Supplementary Information

Jagged1-Notch1-deployed tumor perivascular niche promotes breast cancer stem cell phenotype through Zeb1

Jiang et al.

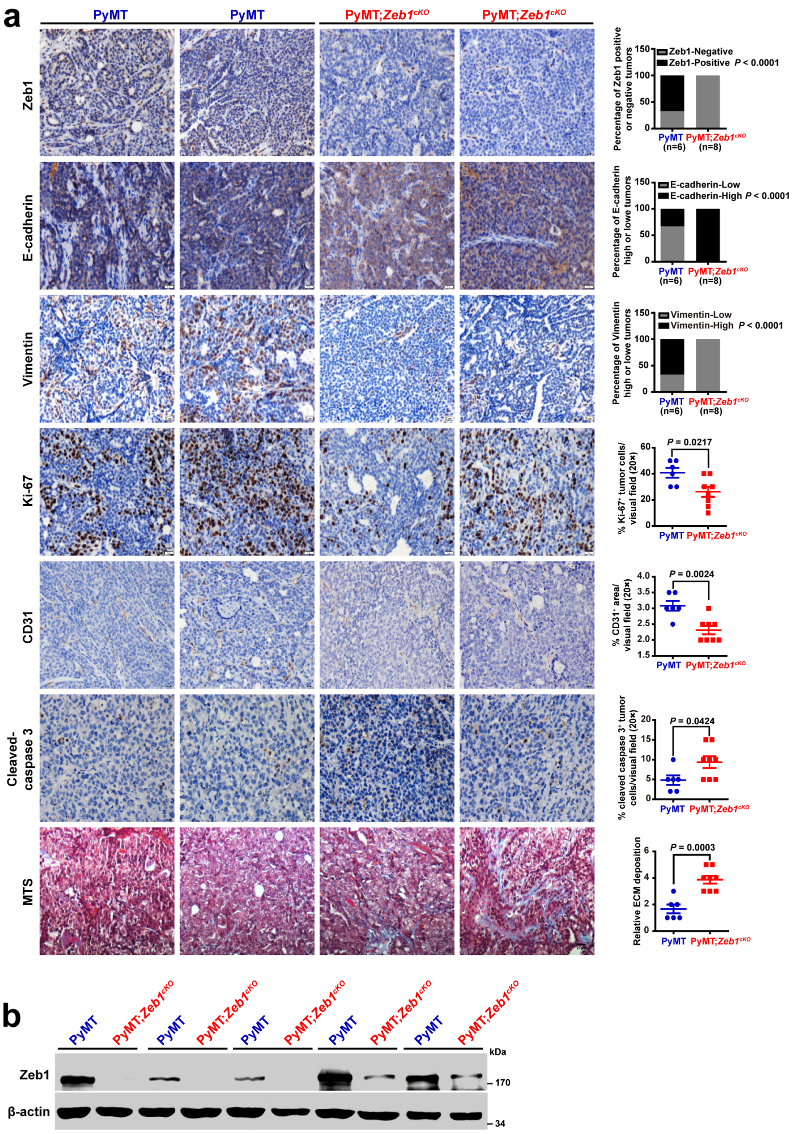

Supplementary Figure 1: Characterization of differentiation markers in PyMT and PyMT;*Zeb1^{cKO}* tumors. (**a**) Representative images of immunohistochemical staining of Zeb1, E-cadherin, Vimentin, Ki-67, CD31, and cleaved-caspase 3 in breast cancer tissue, and quantifications of the indicated markers. Specific blue MTS (Masson's trichrome staining) labels collagen fibers (n = 6 PyMT, 8 PyMT;*Zeb1^{cKO}*). Indicated *P* values were calculated using two-sided Fisher's exact test or two-tailed unpaired Student's *t*-test. Scale bars, 20 µm. (**b**) Protein levels for Zeb1 in EpCAM^{+} tumor cells (n = 5 PyMT, 5 PyMT;*Zeb1^{cKO}*). Data are presented as mean ± SEM in **a**. Dots depict individual samples in **a**. Data are representative of at least five (**a**, **b**) independent experiments.

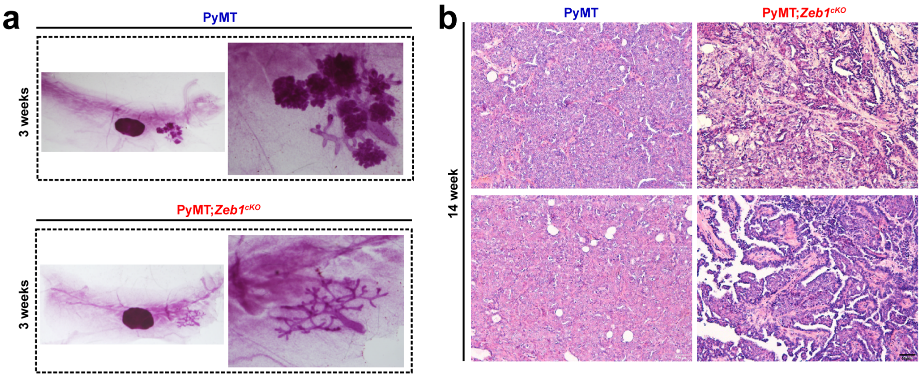

Supplementary Figure 2: Histological analysis of PyMT and PyMT;*Zeb1^{cKO}* tumors. (**a**) Representative alum carmine-stained whole mount mammary outgrowths from PyMT and PyMT;*Zeb1^{cKO}* mice at the indicated ages (n = 5 PyMT, 5 PyMT;*Zeb1^{cKO}*). (**b**) Representative haematoxylin and eosin (HE)-stained sections for the grading of the respective tumors (n = 5 PyMT, 5 PyMT;*Zeb1^{cKO}*). Scale bars, 50 µm.

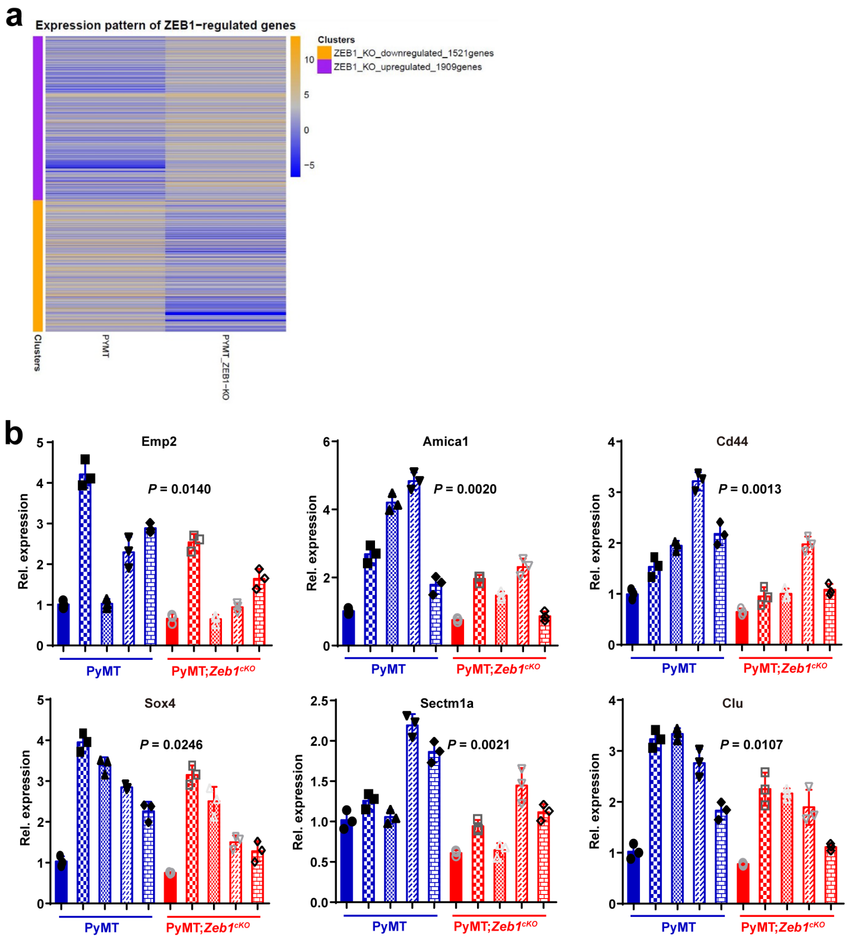

Supplementary Figure 3: Depletion of *Zeb1* affects the transcriptomes of PyMT;*Zeb1*^{cKO} and PyMT cells. (**a**) Differentially expressed genes in global gene-expression analysis from PyMT;*Zeb1*^{cKO} vs. PyMT cells. (**b**) Relative mRNA levels for stemness-enrichment genes (n = 5 PyMT, 5 PyMT;*Zeb1*^{cKO}). Indicated *P* values were calculated using two-tailed unpaired Student's *t*-test in **b**. Data are presented as mean ± SEM in **b**. Data are representative of five (**b**) independent experiments.

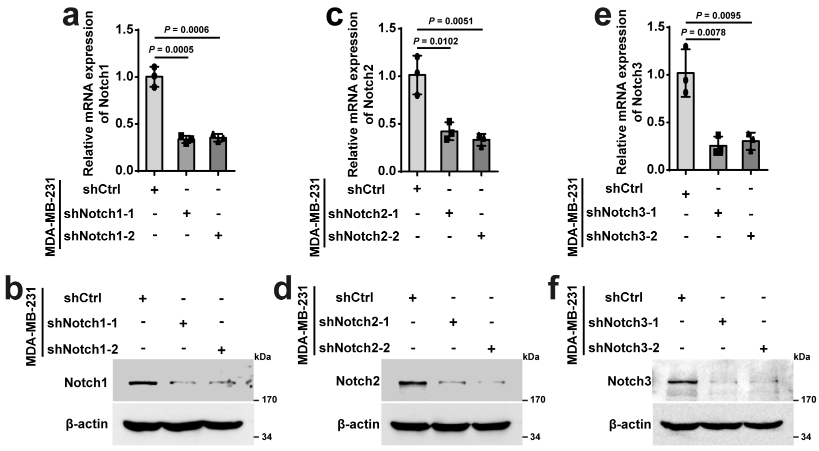

Supplementary Figure 4: The knockdown efficiency of Notch1, Notch2 and Notch3 in MDA-MB-231 cells. (**a-f**) MDA-MB-231 cells were stably transfected with the specific shRNA targeting Notch1, Notch2 or Notch3. The expression of Notch1, Notch2 and Notch3 were verified by quantitative PCR (**a**, **c**, **e**) and immunoblotting (**b**, **d**, **f**) and normalized to the levels of β-actin. Indicated *P* values were calculated using two-tailed unpaired Student's *t*-test in **a**, **c**, **e**. Data are presented as mean ± SEM in **a**, **c**, **e**. Data are representative of three (**a-f**) independent experiments.

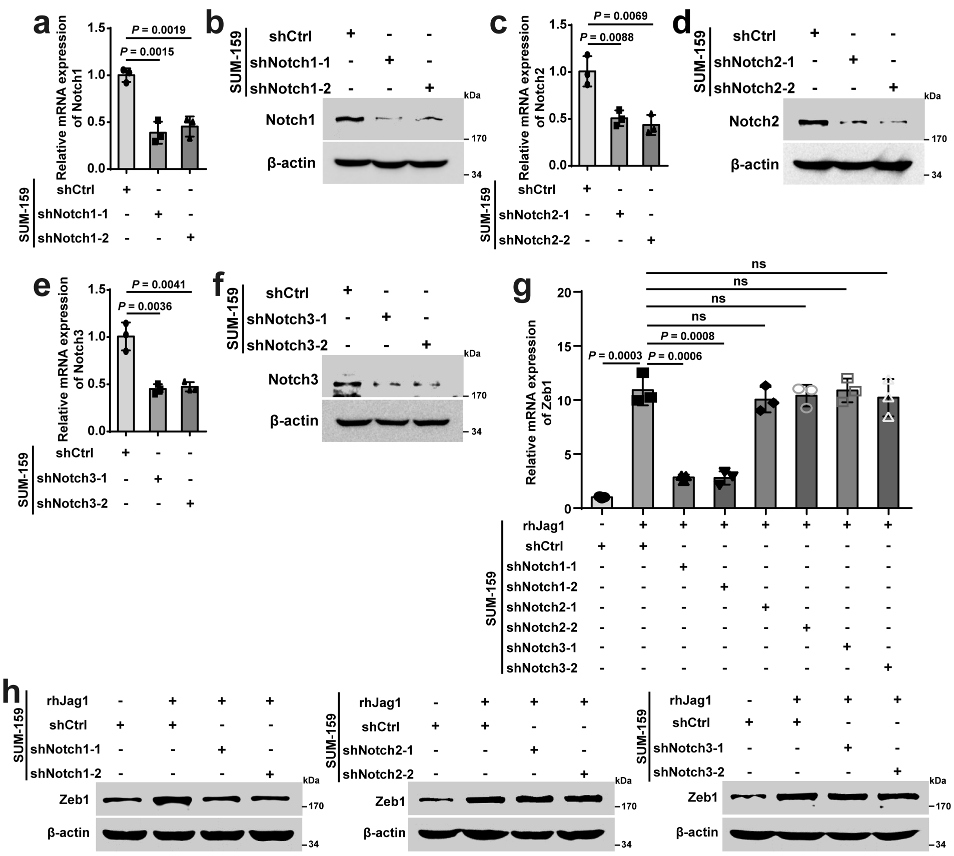

Supplementary Figure 5: Notch1 activation upregulates Zeb1 expression in SUM-159 cells. (**a**-**f**) SUM-159 cells were stably transfected with the specific shRNA targeting Notch1, Notch2 or Notch3. The expression of Notch1, Notch2 and Notch3 were verified by quantitative PCR (**a**, **c**, **e**) and immunoblotting (**b**, **d**, **f**) and normalized to the levels of β-actin. (**g**-**h**) Relative mRNA (**g**) and protein (**h**) levels for Zeb1 in shNotch1-, shNotch2- or shNotch3-interfered SUM-159 cells after treatment with rhJag1. Indicated *P* values were calculated using two-tailed unpaired Student's *t*-test in **a**, **c**, **e**, **g**. Data are presented as mean ± SEM in **a**, **c**, **e**, **g**. Data are representative of three (**a**-**h**) independent experiments.

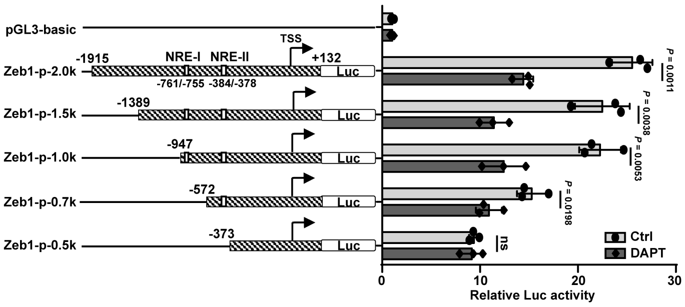

Supplementary Figure 6: Notch1 inhibition decreases the transcriptional activity of Zeb1. Luciferase assays for wild-type (-1915/+132) or truncated promoters of Zeb1 in MDA-MB-231 cells after treatment with DAPT. Indicated *P* values were calculated using two-tailed unpaired Student's *t*-test. Data are presented as means ± SEM. Data are representative of three independent experiments.

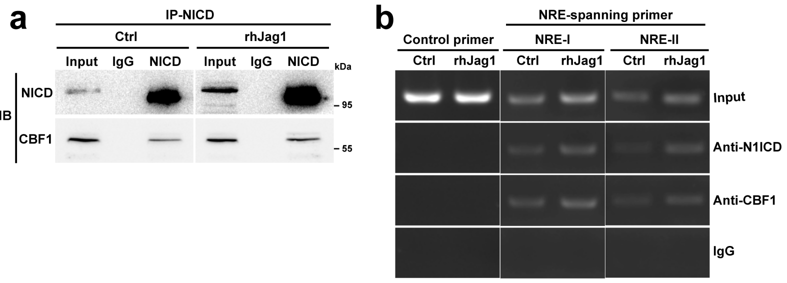

Supplementary Figure 7: NICD recruits to the Zeb1 promoter in an NRE dependent manner by forming a complex with CBF1. (**a**) The interactions between NICD and CBF1 protein were analyzed by co-immunoprecipitation in MDA-MB-231 in the presence or absence of rhJag1. (**b**) The association of NICD and CBF1 with the Zeb1 promoter was analyzed by ChIP assay. The amplified sequences of the Zeb1 promoter fragment containing NRE-I and NRE-II elements are shown. Input DNA amounts were confirmed by equal loading of chromatin. Data are representative of three (**a**, **b**) independent experiments.

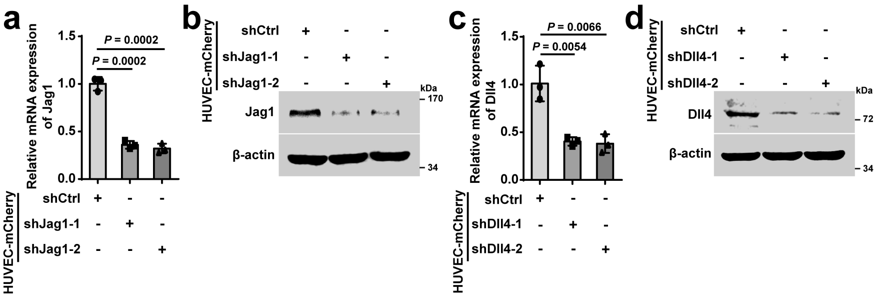

Supplementary Figure 8: The knockdown efficiency of Jag1 and Dll4 in HUVEC-mCherry. (**a-d**) HUVEC-mCherry were stably transfected with the specific shRNA targeting Jag1 or Dll4. The expression of Jag1 and Dll4 were verified by quantitative PCR (**a**, **c**) and immunoblotting (**b**, **d**) and normalized to the levels of β-actin. Indicated *P* values were calculated using two-tailed unpaired Student's *t*-test in **a**, **c**. Data are presented as mean ± SEM in **a**, **c**. Data are representative of three (**a-d**) independent experiments.

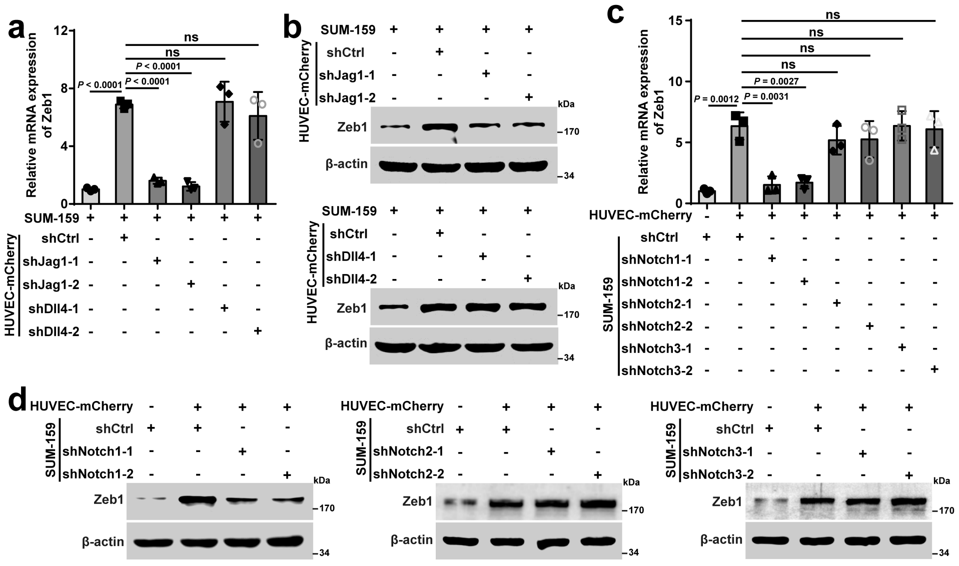

Supplementary Figure 9: Endothelial Jag1 induces Notch1 activation in SUM-159 cells. (**a**-**b**) Relative mRNA (**a**) and protein (**b**) levels for Zeb1 in SUM-159 cells that were cocultured with shJag1- or shDll4-interfered HUVEC-mCherry. (**c**-**d**) Relative mRNA (**c**) and protein (**d**) levels for Zeb1 in shNotch1-, shNotch2- or shNotch3-interfered SUM-159 cells that were cocultured with HUVECs. Indicated *P* values were calculated using two-tailed unpaired Student's *t*-test in **a**, **c**. Data are presented as mean ± SEM in **a**, **c**. Data are representative of three (**a**-**d**) independent experiments.

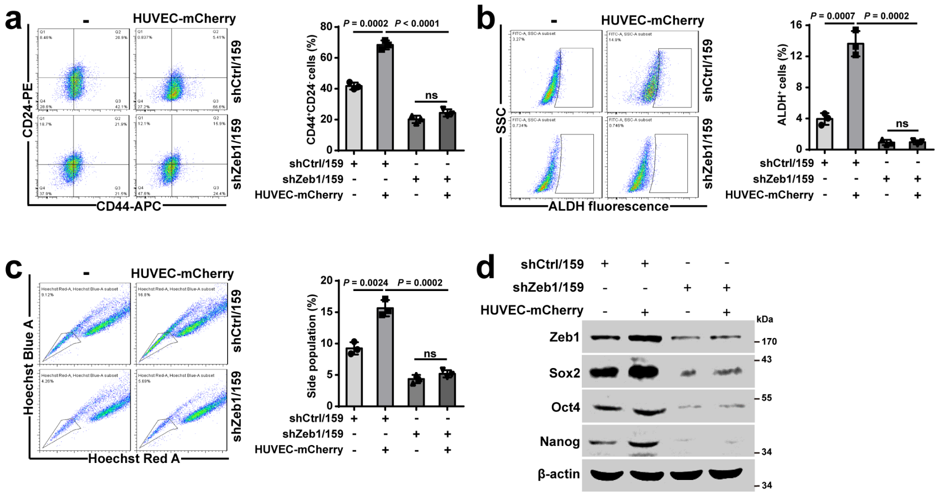

Supplementary Figure 10: Endothelial Jag1 induced-activation Notch1 contributes to CSC phenotypes through a Zeb1-dependent mechanism in SUM-159 cells. (**a-c**) Flow cytometry analysis for (**a**) CD44^{+}CD24^{-} population, (**b**) ALDH activity and (**c**) side-population assay in shCtrl- or shZeb1-interfered SUM-159 cells that were cocultured with HUVEC-mCherry. (**d**) Protein levels for Zeb1, Sox2, Oct4 and Nanog in shCtrl- or shZeb1-interfered SUM-159 cells that were cocultured HUVEC-mCherry. Indicated P values were calculated using two-tailed unpaired Student's t-test in **a-c**. Data are presented as mean ± SEM in **a-c**. Data are representative of three (**a-d**) independent experiments.

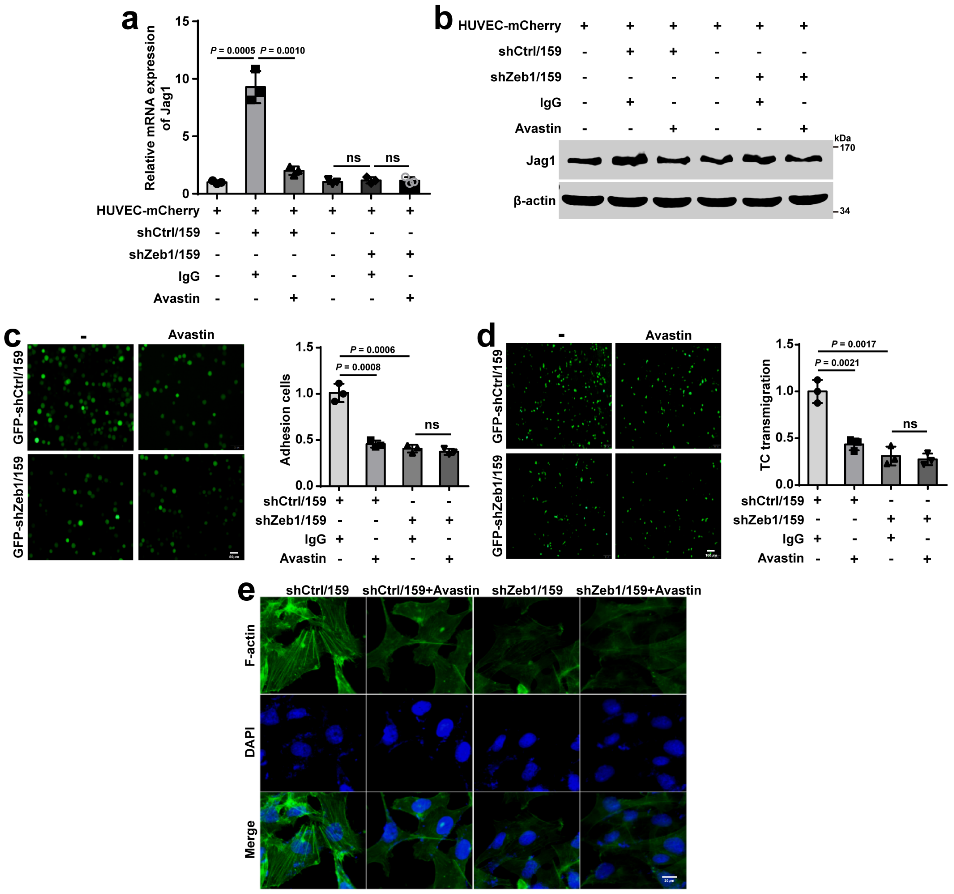

Supplementary Figure 11: Depletion of Zeb1 impairs tumor-endothelial cell interaction through VEGFA in SUM-159 cells. (**a-b**) Relative mRNA (**a**) and protein (**b**) levels for Jag1 in HUVEC-mCherry that were cocultured with shCtrl- or shZeb1-interfered SUM-159 cells in the presence of Avastin. (**c**) Tumor cell (shCtrl- or shZeb1-interfered SUM-159) adhesion to HUVEC monolayers in the presence of Avastin. Scale bar, 50 µm. (**d**) Tumor cell (shCtrl- or shZeb1-interfered SUM-159) transmigration through HUVEC monolayers seeded on transwell inserts in the presence of Avastin. Scale bar, 100 µm. (**e**) Immunofluorescent staining for Phalloidin of F-actin filaments in HUVECs after treatment with conditioned media derived from shCtrl- or shZeb1-interfered SUM-159 in the presence of Avastin. Scale bar, 20 µm. Indicated *P* values were calculated using two-tailed unpaired Student's *t*-test in **a**, **c**, **d**. Data are presented as mean ± SEM in **a**, **c**, **d**. Data are representative of three (**a-e**) independent experiments.

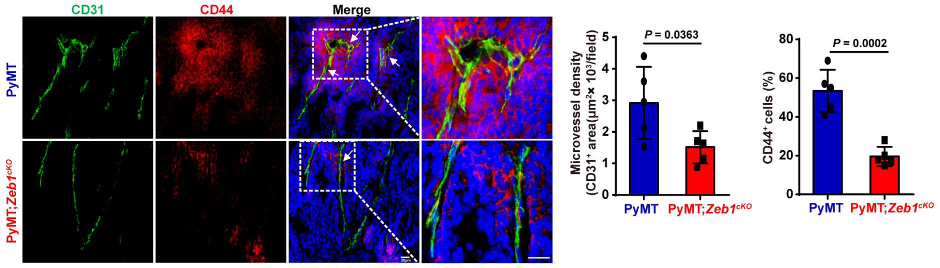

Supplementary Figure 12: Depletion of Zeb1 subverts the tumor vascular niche. Immunofluorescence staining for CD31 and CD44 in breast cancer tissue (n = 5 PyMT, 5 PyMT;*Zeb1*^{cKO}). The highlighted regions in the left panels are enlarged in the right panels. Scale bars, 20μm. Indicated *P* values were calculated using two-tailed unpaired Student's *t*-test. Data are presented as mean ± SEM. Dots depict individual samples. Data are representative of five independent experiments.

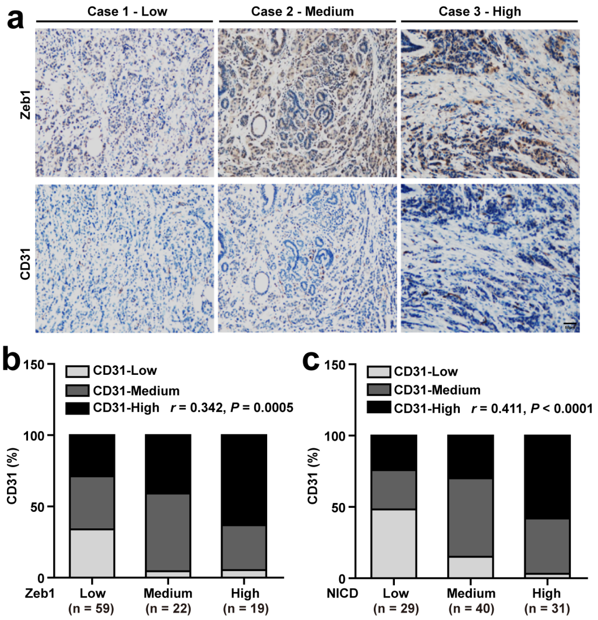

Supplementary Figure 13: Zeb1 expression is positively correlated with tumor angiogenesis in aggressive human breast cancer. (**a**) Representative images of immunohistochemical staining of Zeb1 and CD31 in serial sections of the same tumor (n = 100). Scale bars, 50 μm. (**b**) A positive correlation between the expression of Zeb1 and CD31 in the 100 human breast cancer samples. (**c**) A positive correlation between the expression of NICD and CD31 in breast cancer. Indicated *P* values were calculated using Spearman's rank correction test in **b**, **c**.

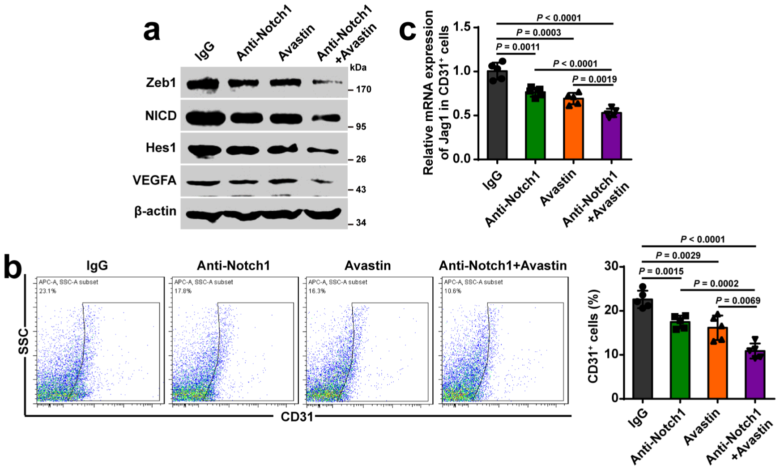

Supplementary Figure 14: Combinatorial treatment of anti-Notch1 and Avastin synergistically reduces tumor angiogenesis. (**a**) Protein levels for Zeb1, NICD, VEGFA and Hes1 in EpCAM$^+$ tumor cells from tumor tissue (n = 5). (**b**) Flow cytometry analysis for CD31$^+$ endothelial cell fraction; n = 5. (**c**) Relative mRNA levels for Jag1 in CD31$^+$ endothelial cells; n = 5. Indicated *P* values were calculated using two-tailed unpaired Student's *t*-test in **b**, **c**. Data are presented as mean ± SEM in **b**, **c**.

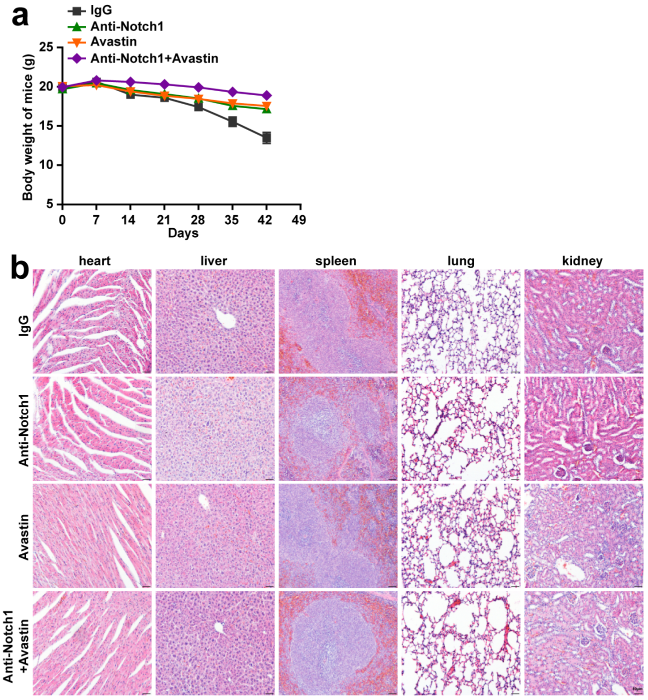

Supplementary Figure 15: Combinatorial treatment of anti-Notch1 and Avastin synergistically has no significantly adverse effects. (**a**) Body weight of BALB/c mice coinjected with MDA-MB-231 and HUVECs following treatment with IgG, anti-Notch1, Avastin, or both anti-Notch1 and Avastin; n = 5. (**b**) Representative haematoxylin and eosin (HE)-stained sections of heart, liver, spleen, lung and kidney from mice with the indicated treatment; n = 5. Scale bars, 50 μm.

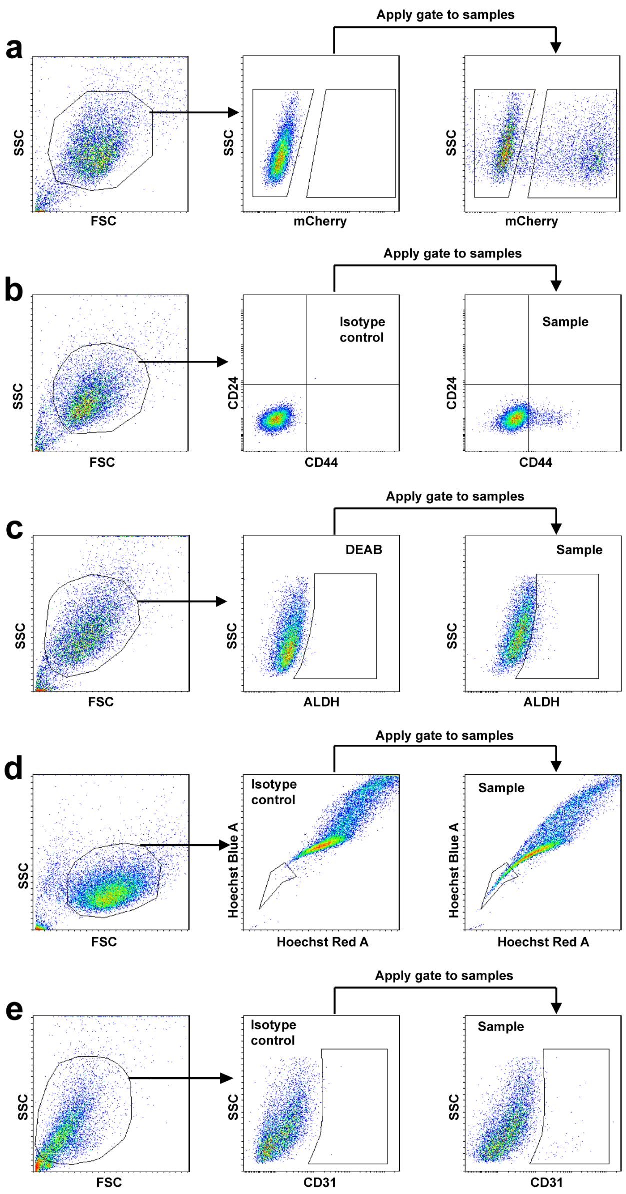

Supplementary Figure 16: Gating strategy for flow cytometer. Gating strategies of flow cytometry analysis for HUVEC-mCherry (**a**), $CD44^{+}CD24^{-}$ population (**b**), $ALDH^{+}$ cells (**c**), Side population (**d**) and $CD31^{+}$ cells (**e**). SSC = Side Scatter, FSC = Forward Scatter.

Supplementary Table 1: Zeb1 promoter activity in response to siRNA interference of human growth factors and receptors.

(*P* value vs. the respective control by two-tailed unpaired Student's *t*-test)

	Gene name	Abbreviation	Fold-change	*P* value
**Downregulated Genes**	NOTCH1	NOTCH1	0.19	0.001
	Wnt Family Member 7A	WNT7A	0.57	0.004
	Wnt Family Member 3	WNT3	0.60	0.009
	Wnt Family Member 4	WNT4	0.62	0.038
	C-C Motif Chemokine Ligand 18	CCL18	0.66	0.036
	Interleukin 18	IL18	0.69	0.030
	Interleukin 1 Beta	IL1B	0.70	0.042
	Wnt Family Member 3A	WNT3A	0.74	0.024
	C-C Motif Chemokine Ligand 21	CCL21	0.84	0.017
	Interleukin 1 Family Member 10 (Theta)	IL1F10	0.86	0.033
	Interleukin 36, Alpha	IL1F6	0.88	0.028
**Upregulated Genes**	Interleukin 13	IL13	2.69	0.000
	Intercellular Adhesion Molecule 2	ICAM2	2.27	0.000
	Interleukin 6	IL6	2.12	0.000
	Interleukin 23 Subunit Alpha	IL23A	2.01	0.002
	Interleukin 5	IL5	2.00	0.000
	Interleukin 1 Receptor Antagonist	IL1RN	1.92	0.010
	Interleukin 3	IL3	1.83	0.010
	Fibroblast Growth Factor 9	FGF9	1.72	0.001
	Intercellular Adhesion Molecule 4	ICAM4	1.71	0.003
	Transforming Growth Factor Beta 3	TGFB3	1.67	0.001
	Interleukin 4	IL4	1.57	0.000
	Transforming Growth Factor Beta 1	TGFB1	1.49	0.000
	Transforming Growth Factor Beta 2	TGFB2	1.49	0.007
	Transforming Growth Factor Beta Induced	TGFBI	1.46	0.041
	Interleukin 36 Receptor Antagonist	IL1F5	1.45	0.003
	Interleukin 36, Beta	IL1F8	1.44	0.004
	Tumor Necrosis Factor Superfamily Member 11	TNFSF11	1.27	0.021
	Interleukin 17A	IL17A	1.22	0.029
	Interleukin 37	IL1F7	1.22	0.036
	Fibroblast Growth Factor 5	FGF5	1.13	0.003
	C-C Motif Chemokine Ligand 4	CCL4	1.07	0.004

Supplementary Table 2: List of antibodies with their sources and experimental conditions.

Protein	Species	Application	Manufacturer	Catalog No.	Dilution
anti-ZEB1	Rabbit	IHC	Abcam	ab87280	1:100
		IB	Santa Cruz	sc-25388	1:1000
anti-NICD	Rabbit	IB	CST	#4147	1:1000
		IP			1:200
		ChIP			
		IF/IHC	Abcam	ab8925	1:100
anti-CBF-1	Rabbit	IP	Abcam	ab25949	5µg/ml
		CHIP			2ug
	Mouse	IB	Proteintech	66132-1-Ig	1µg/ml
anti-JAG1	Goat	WB	Santa Cruz	sc-6011	1:500
anti-NOTCH1	Rabbit	WB	Proteintech	20687-1-AP	1:1000
anti-NOTCH 2	Rabbit	WB	CST	#5732	1:1000
anti-NOTCH 3	Rabbit	WB	Proteintech	55114-1-AP	1:1000
anti-DLL4	Rabbit	WB	Proteintech	21584-1-AP	1:1000
anti-α-SMA	Rabbit	IF	Proteintech	14395-1-AP	1:100
anti-CD31	Rat	IF	BD	553370	1:100
anti-CD31	Rabbit	IHC	Abcam	ab28364	1:100
anti-CD31-APC	Rat	FC	BD	551262	1:100
anti-EpCAM-APC	Rat	FC	BD	563478	1:100
anti-ALDH1A1	Mouse	IHC	Santa Cruz	sc-374149	1:100
		IF			
anti-CD44-APC	Mouse	FC	BD	559942	1:100
anti-CD24-PE	Mouse	FC	BD	555428	1:100
anti-CD44-PE	Rat	FC	BD	561860	1:100
anti-CD24-APC	Rat	FC	BD	562349	1:100
Anti-CD44	Rabbit	IF	Invitrogen	701406	1:100
anti-SOX2	Rabbit	IB	CST	#3579	1:1000
anti-OCT4	Rabbit	IB	CST	#2750	1:1000
anti-NANOG	Rabbit	IB	CST	#4903	1:1000
anti-VEGFA	Rabbit	IHC	Proteintech	19003-1-AP	1:100
anti-HES1	Rabbit	IB	CST	#11988	1:1000
anti-E-cadherin	Mouse	IF	CST	#14472	1:50
		IHC			1:100
anti-Vimentin	Rabbit	IF/IHC	CST	#5741	1:100
anti-Ki-67	Rabbit	IHC	CST	#9027	1:400
anti-β-actin	Mouse	IB	Santa Cruz	sc-47778	1:1000
anti-Phalloidin-FITC		IF	Sigma	P5282	5µg/ml
Dextran			Invitrogen	D1822	10mg/ml

AlexaFluor® 594-conjugated Goat anti-rat IgG	Goat	IF	Abcam	ab150160	1:200
AlexaFluor® 488-conjugated Goat anti-rat IgG	Goat	IF	Abbkine	A23240	1:200
AlexaFluor® 594-conjugated Goat anti-rabbit IgG	Goat	IF	Abbkine	A23420	1:200
AlexaFluor® 594-conjugated Goat anti-mouse IgG	Goat	IF	Abbkine	A23410	1:200

IHC: Immunohistochemistry; IB: Immunoblotting; IF: Immunofluorescence; IP: Immunoprecipitation; ChIP: Chromatin immunoprecipitation, FC: Flow cytometry

Supplementary Table 3: Sequences of the oligonucleotides for shRNA, mice genotyping, real-time PCR, and site-mutagenesis.

Construction of shRNA vector	
shZeb1-1	5’-CGGCGCAATAACGTTACAAAT-3’
shZeb1-2	5’-GGCGCAATAACGTTACAAA-3’
shZeb1-3	5’-CCTCTCTGAAAGAACACATTA-3’
shJag1-1	5’-CGCGTGACCTGTGATGACTACTACT-3’
shJag1-2	5’-TAAGAGTTCAGAGGCGGCCTCTGAA-3’
shJag1-3	5’- GGACATCGATTATTGTGAGCCTAAT-3’
shDll4-1	5’-GGGCTGTCATGAACAGAAT-3’
shDll4-2	5’-GCTGGTCTTCAGAAATCAA-3’
shNotch1-1	5’- GGAGCATGTGTAACATCAA-3’
shNotch1-2	5’- GGGACATCACGGATCATAT-3’
shNotch2-1	5’- GGGTATGAAATCATCCTTAT-3’
shNotch2-2	5’-GCTCTGATGTTGTGGTTTA-3’
shNotch3-1	5’-GCAGATGTATGCATTCCTT-3’
shNotch3-2	5’-GCTTGGGAAATCAGCCTTA-3’
Construction of *Zeb1* promoter	
-373-forward	5’-TATTACTCATTCCGCTCTAC-3’
-572-forward	5’-AAGGTCCTGCACGGCGATGA-3’
-947-forward	5’-TACTATGTGTCATTGTGCTA-3’
-1389-forward	5’-CCAAGCAAAGCAAGGACACC-3’
-1915-forward	5’-GGATGAAGGCAGATTATGTA-3’
reverse	5’-GGCTGTCGGAGTTGGAAAGGTAAAG-3’
Site-Directed Mutagenesis of ZEB1 promoter	
NRE-I forward	5’-GATTTAGGTTTCCTTCCTGCTTAACACCTCCTTCGAATAAGGAAAC-3’
NRE-I reverse	5’-GTTTCCTTATTCGAAGGAGGTG**TT**AAGCAGGAAGGAAACCTAAATC-3’
NRE-II forward	5’-TAGAGCGGAATGAGTAATAAACACACGGTGCTTGTCTCAC-3’
NRE-II reverse	5’-GTGAGACAAGCACCGTGTG**TT**TATTACTCATTCCGCTCTA-3’
Mice genotyping	
PyMT-F	5’-GGAAGCAAGTACTTCACAAGGG-3’
PyMT-R	5’-GGAAAGTCACTAGGAGCAGGG-3’
Cre-F	5’-ATTTGCCTGCATTACCGGTC-3’
Cre-R	5’-ATCAACGTTTTCTTTTCG G-3’
Zeb1-LoxP-F	5’-GTCTATCCAGAATCTTCCCATGAC-3’
Zeb1-LoxP-R	5’-CTGTCAATCTCTGGCCTCTACATG-3’
Rea-Time PCR	
human Zeb1 forward	5’-CAGCTTGATACCTGTGAATGGG-3’

human Zeb1 reverse	5’-TATCTGTGGTCGTGTGGGACT-3’
human Jag1 forward	5’-AATGGTTATCGCTGTATCTG-3’
human Jag1 reverse	5’-TCACTGGCACGGTTGTAG-3’
human Dll4 forward	5’-GGATGAGCAAACCAGCAC-3’
human Dll4 reverse	5’-ATGACAGCCCGAAAGACA-3’
human Notch1 forward	5’-ATGTTGGAGAAGGGAAGTTG-3’
human Notch1 reverse	5’-GCAGGTGATGCTGGTGGA-3’
human Notch2 forward	5’-TTGATGATTGTGCCTTCG-3’
human Notch2 reverse	5’-CCTTTGTAGCCTTGTGGG-3’
human Notch3 forward	5’-GGATTGCCGTCAGTGGA-3’
human Notch3 reverse	5’-TCAGGTCGGAGATGATGCT-3’
mouse Amica1 forward	5’-CCGCAACCGTGTAGACCTG-3’
mouse Amica1 reverse	5’-GATGCCCTGCTGACCCTTAT-3’
mouse Cd44 forward	5’-TTCATAAGAACAGGGATTGC-3’
mouse Cd44 reverse	5’-AAAAACTGGAAAAAGAAAGG-3’
mouse Emp2 forward	5’-GAGTCTGAGTGGCTTTAGGG-3’
mouse Emp2 reverse	5’-AAGGCAACAGGTGATCGTTT-3’
mouse Sectm1a forward	5’-GCTTGCTGGAGAAGGGAC-3’
mouse Sectm1a reverse	5’-ACATCGGTGAAGGTGTTAGA-3’
mouse Clu forward	5’-AGCCGTGCGGAATGAGATAG-3’
mouse Clu reverse	5’-TATTGGATTAGTTGCGTGGG-3’
mouse Sox4 forward	5’-AAGAAAAGGAAGAAAAAGT-3’
mouse Sox4 reverse	5’-CCACCAACATCAATAACAA-3’
Quantitative ChIP	
NRE-I(-863) forward	5’-AACTGCTACATTGTTAAT-3’
NRE-I(-698) reverse	5’-AATATCAAGAAAGTCGTC-3’
NRE-II(-521) forward	5’-GATACCTTAGCTCTGAGT-3’
NRE-II(-326) reverse	5’- AGAAGTTCCGCTTGCCAG-3’
