# Supplementary material for: Jagged1-Notch1-deployed tumor perivascular niche promotes breast cancer stem cell phenotype through Zeb1
Source: Nat Commun. 2020 Oct 12;11:5129. doi: 10.1038/s41467-020-18860-4 (PMC7552407; doi:10.1038/s41467-020-18860-4)
Supplement: Supplementary file 3 — Reporting Summary [file 41467_2020_18860_MOESM3_ESM.pdf]

nature research

Corresponding author(s): Shuang Yang

Last updated by author(s): Sep 2, 2020

Reporting Summary

Nature Research wishes to improve the reproducibility of the work that we publish. This form provides structure for consistency and transparency in reporting. For further information on Nature Research policies, see Authors & Referees and the Editorial Policy Checklist .

Statistics

For all statistical analyses, confirm that the following items are present in the figure legend, table legend, main text, or Methods section.

n/a	Confirmed	
☐	☒	The exact sample size (*n*) for each experimental group/condition, given as a discrete number and unit of measurement
☐	☒	A statement on whether measurements were taken from distinct samples or whether the same sample was measured repeatedly
☐	☒	The statistical test(s) used AND whether they are one- or two-sided<br>*Only common tests should be described solely by name; describe more complex techniques in the Methods section.*
☒	☐	A description of all covariates tested
☐	☒	A description of any assumptions or corrections, such as tests of normality and adjustment for multiple comparisons
☐	☒	A full description of the statistical parameters including central tendency (e.g. means) or other basic estimates (e.g. regression coefficient) AND variation (e.g. standard deviation) or associated estimates of uncertainty (e.g. confidence intervals)
☐	☒	For null hypothesis testing, the test statistic (e.g. *F*, *t*, *r*) with confidence intervals, effect sizes, degrees of freedom and *P* value noted<br>*Give P values as exact values whenever suitable.*
☒	☐	For Bayesian analysis, information on the choice of priors and Markov chain Monte Carlo settings
☒	☐	For hierarchical and complex designs, identification of the appropriate level for tests and full reporting of outcomes
☐	☒	Estimates of effect sizes (e.g. Cohen's *d*, Pearson's *r*), indicating how they were calculated

Our web collection on statistics for biologists contains articles on many of the points above.

Software and code

Policy information about availability of computer code

Data collection	BD FACSArial ⁴ was used to collect flow cytometric data. qPCR data was collected using Roche LightCycler 480. Immunofluorescence images were acquired with FV1000 confocal microscope (Olympus). Immunohistochemical images were taken using BX53 microscope (Olympus). Densitometric analysis of western blot imaging was performed by G:Box Chemi XRQ gel doc system (Syngene).
Data analysis	The data were analyzed using Flow Jo software (version 7.6), GraphPad Prism Version 5 and 7, ImageJ (1.42q), SPSS 22.<br>The tumor-initiating cell frequency was determined using the ELDA webtool.

For manuscripts utilizing custom algorithms or software that are central to the research but not yet described in published literature, software must be made available to editors/reviewers. We strongly encourage code deposition in a community repository (e.g. GitHub). See the Nature Research guidelines for submitting code & software for further information.

Data

Policy information about availability of data

All manuscripts must include a data availability statement. This statement should provide the following information, where applicable:

- Accession codes, unique identifiers, or web links for publicly available datasets
- A list of figures that have associated raw data
- A description of any restrictions on data availability

RNA-seq data that support the findings of this study have been deposited in NCBI_SRA with accession number PRJNA511636 (https://www.ncbi.nlm.nih.gov/sra/?term=PRJNA511636). All the other data supporting the findings of this study are available within the article and its Supplementary Information files and from the corresponding author upon reasonable request. Data used to generate the figures are available in the Source Data file provided with this paper. Source data are provided with this paper.

Field-specific reporting

Please select the one below that is the best fit for your research. If you are not sure, read the appropriate sections before making your selection.

- [x] Life sciences
- [ ] Behavioural & social sciences
- [ ] Ecological, evolutionary & environmental sciences

For a reference copy of the document with all sections, see nature.com/documents/nr-reporting-summary-flat.pdf

Life sciences study design

All studies must disclose on these points even when the disclosure is negative.

Sample size	Sample size was chosen based on our prior studies using the same types of assays, as well as published literature, to ensure statistically significant results. For student's t test, at least 3 biological repeats was used to calculate p value. The sample size is commonly acceptable in biological experiment. All sample size was revealed in the sections of Materials and Methods or Figure Legends.
Data exclusions	No data were excluded from the analyses.
Replication	Experiments were repeated with at least three biologically independent studies for all results presented in the main manuscript. All replication attempts were successful.
Randomization	Allocation was random.
Blinding	No blinding, as the same investigator performed most experiments and analyzed the data. For the IHC, both staining and analysis was completed in a blinded manner.

Reporting for specific materials, systems and methods

We require information from authors about some types of materials, experimental systems and methods used in many studies. Here, indicate whether each material, system or method listed is relevant to your study. If you are not sure if a list item applies to your research, read the appropriate section before selecting a response.

Materials & experimental systems

n/a	Involved in the study	
[ ]	[x]	Antibodies
[ ]	[x]	Eukaryotic cell lines
[x]	[ ]	Palaeontology
[ ]	[x]	Animals and other organisms
[ ]	[x]	Human research participants
[x]	[ ]	Clinical data

Methods

n/a	Involved in the study	
[x]	[ ]	ChIP-seq
[ ]	[x]	Flow cytometry
[x]	[ ]	MRI-based neuroimaging

Antibodies

Antibodies used	Descriptions of antibodies used (including concentration, supplier name, clone name and catalog number) can be found in supplementary information section 'Antibodies'.
Validation	All used antibodies were validated commercially. Certificated of analysis for the approved applications by the manufacturer are available on the company websites.

Eukaryotic cell lines

Policy information about cell lines

Cell line source(s)	MDA-MB-231, HUVEC, SUM-159 and HEK293T were obtained from ATCC.
Authentication	Cell line authentication was not performed as cells were not listed in the commonly misidentified category.
Mycoplasma contamination	All cell lines used in this study tested negative for mycoplasma before their use.
Commonly misidentified lines (See ICLAC register)	No commonly misidentified cell line were used in the study.

Animals and other organisms

Policy information about studies involving animals; ARRIVE guidelines recommended for reporting animal research

Laboratory animals	Mice were housed under standard specfic-pathogen-free (SPF) conditions.<br>Female mice, NOD-SCID strain (for cell lines), 6-8 weeks old.<br>Zeb1 fl/fl mice were generated from knockout mammary epithelial cells for Zeb1 (C57BL/6) in our laboratory. MMTV-PyMT (FVB) mice and MMTV-Cre (C57BL/6) mice were purchased from model animal research center of Nanjing University. All mice were kept with littermates used in all experiments, and gender-matched littermate controls were used in all experiments.<br>All mice were housed in a temperature-controlled room (22 ± 2 °C) with 40-60% humidity, with a light/dark cycle of 12h/12h.
Wild animals	No wild animals were included.
Field-collected samples	No field-collected samples were included.
Ethics oversight	Mice were handled in accordance with protocols approved by the Animal Care and Use Committees of Medical College of Nankai University and Institute of Radiation Medicine of Chinese Academy of Medical Science.

Note that full information on the approval of the study protocol must also be provided in the manuscript.

Human research participants

Policy information about studies involving human research participants

Population characteristics	A total of 175 breast cancer patient samples were obtained from the General Hospital of the People's Liberation Army (PLAGH, Beijing, China) along with pathological information. All patients had been histologically confirmed to have invasive ductal carcinoma of the breast and were recruited by the same department.
Recruitment	Patients were not actively recruited. Invasive ductal breast cancer samples were obtained after written informed consent following institutional guidelines of PLAGH. Histological confirmation of invasive ductal breast cancer was obtained from the Department of Pathology at the hospital.
Ethics oversight	This study was approved by the institutional ethics committees at PLAGH and Medical College of Nankai University. All patients provided informed consent according to the latest version of the Helsinki Declaration on human research ethics.

Note that full information on the approval of the study protocol must also be provided in the manuscript.

Flow Cytometry

Plots

Confirm that:

- [x] The axis labels state the marker and fluorochrome used (e.g. CD4-FITC).
- [x] The axis scales are clearly visible. Include numbers along axes only for bottom left plot of group (a 'group' is an analysis of identical markers).
- [x] All plots are contour plots with outliers or pseudocolor plots.
- [x] A numerical value for number of cells or percentage (with statistics) is provided.

Methodology

Sample preparation	Sample preparation protocol is describe in Methods section 'primary cell lines' and 'Flow cytometry'.
Instrument	FACSAria II (BD Biosciences)
Software	Flow cytometry data were collected via Diva (BD Biosciences) and analyzed by FlowJo V7.6.
Cell population abundance	Gated cells from all samples were between 70~90% of all events.
Gating strategy	The starting cell populations were gated from FSC/SSC plot.

- [x] Tick this box to confirm that a figure exemplifying the gating strategy is provided in the Supplementary Information.
